# Sensor-Based Assessment of Morning Prospective Memory in Narcolepsy Type 1: Evidence from Children and Adult Cohorts

**DOI:** 10.3390/s26092681

**Published:** 2026-04-26

**Authors:** Lorenzo Tonetti, Sara Giovagnoli, Martina Gnazzo, Miranda Occhionero, Fabio Pizza, Giuseppe Plazzi, Vincenzo Natale

**Affiliations:** 1 Department of Psychology “Renzo Canestrari”, University of Bologna, 40127 Bologna, Italy; sara.giovagnoli@unibo.it (S.G.); miranda.occhionero@unibo.it (M.O.); vincenzo.natale@unibo.it (V.N.); 2 Clinic of Child and Adolescent Neuropsychiatry, Department of Mental and Physical Health and Preventive Medicine, University of Campania “Luigi Vanvitelli”, 80138 Naples, Italy; martignazzo@hotmail.it; 3 IRCCS Institute of Neurological Sciences of Bologna, 40139 Bologna, Italy; fabio.pizza@unibo.it (F.P.); giuseppe.plazzi@unimore.it (G.P.); 4 Department of Biomedical and Neuromotor Sciences (DIBINEM), University of Bologna, 40139 Bologna, Italy; 5 Department of Biomedical, Metabolic and Neural Sciences, University of Modena and Reggio Emilia, 41125 Modena, Italy

**Keywords:** prospective memory, narcolepsy type 1, actigraphy, sleep inertia, children, adults

## Abstract

The aim of this study was to shed light on activity-based prospective memory performance in children and adult patients with narcolepsy type 1 (NT1) during the first sixty minutes after morning awakening. With reference to the children (C) sample, 21 C-NT1 patients and 20 healthy controls (C-HC) were enrolled; as regards adults (A), 31 A-NT1 patients and 51 A-HC were examined. Each participant used an actigraph for one week, being asked to press the event marker button at get-up time (prospective memory task—PMT). Computing the time interval between the get-up time and the moment the event marker button was pushed, no significant differences were observed in the children’s groups. In contrast, in the adult groups, A-NT1 patients had a longer interval than A-HC. Moreover, the rate of compliant performers (those who remembered to push the event-marker button within 2 min of get-up time) and late performers was significantly different in adults only, with a low rate of compliant performers in A-NT1 patients. In C-NT1, a shorter cognitive inertia was associated with higher motor activity after awakening; in contrast, this association was not observed in A-NT1. Overall, the present pattern of results suggests that prospective memory performance is impaired only in NT1 adult patients.

## 1. Introduction

According to the text revision of the third edition of the International Classification of Sleep Disorders (ICSD-3-TR) [1], narcolepsy type 1 (NT1) is a central disorder of hypersomnolence characterized by cataplexy, low hypocretin-1 concentration, and diurnal periods of uncontrollable need to sleep. This last aspect can be interpreted as a consequence of excessive daytime sleepiness, which has been suggested as the cause of cognitive impairments in NT1. However, these had also been observed in fully alert patients [2].

Among the different cognitive domains objectively assessed, the evidence for sustained attention impairments in NT1 is strong, while the results in the memory domain are mixed [2,3]. However, in a recent survey [4], the 55% of NT1 respondents reported memory problems as the most troubling symptom. A potential source of the not completely homogeneous pattern of results may be the setting of the assessment, with a task performed in the laboratory that may not reflect the functioning of the same cognitive process in everyday life. A specific type of memory, highly involved in everyday life, is prospective memory (PM), conceived as the ability to remember to act in the future. An example of a prospective memory task in everyday life that may have important implications for individuals’ health is remembering to take a specific drug at a specific time. The consequences of cognitive impairments, such as poor prospective memory, on therapy adherence in NT1 patients [5] may be serious. 

Despite the potential relevance of prospective memory in NT1 patients, the literature is somewhat scarce. Indeed, few studies have ecologically explored the activity-based prospective memory performance by asking persons to remember to push the event-marker button of the actigraph to signal the get-up time; more in detail, the efficiency of this performance was examined by computing the ratio between the number of times the task was performed and the overall number of recording days multiplied by 100. Tonetti et al. [6], examining adult samples, reported a significantly lower efficiency in NT1 patients (62.6%) compared to healthy controls (HC) (89.5%). Later [7], the same performance was compared among adult patients with a primary sleep disorder—narcolepsy type 1 and primary insomnia—or a comorbid low sleep quality—e.g., attention-deficit hyperactivity disorder. The efficiency of NT1 activity-based prospective memory performance was significantly lower (47.14%) than in primary insomnia (78.50%), attention-deficit hyperactivity disorder (88.45%), and the remaining clinical samples. This pattern of results led the authors to suggest that, while the role of sleep quality in modulating this specific cognitive activity is reduced, NT1 may be characterized by a disease-specific PM deficit. Such an impairment could be explained by the fact that event-based prospective memory, overall similar to activity-based [8], has been reported to be poor in idiopathic REM sleep behaviour disorder [9], which is highly comorbid with NT1 [10]. 

More recently, the activity-based prospective memory performance was not assessed, as previously conducted, in terms of presence or absence. However, its inertia, computed as the time interval between the get-up time and the moment the task was actually performed, has been examined and correlated with motor sleep inertia in the first 60 min after the get-up time in both attention-deficit/hyperactivity disorder [11] and insomnia [12]. On the contrary, to the best of our knowledge, these aspects have not yet been explored in narcolepsy type 1.

The present exploratory study aims to shed light for the first time on peculiarities of activity-based prospective memory performance, computed by measuring the time interval in minutes between the get-up time and the moment the event-marker button of the actigraph was pushed, in children and adult NT1 patients, and to examine its association with motor sleep inertia in the first 60 min after awakening.

## 2. Materials and Methods

### 2.1. Participants

Four different groups were examined in the present study: children patients with NT1 (C-NT1), children healthy controls (C-HC), adult patients with NT1 (A-NT1), and adult healthy controls (A-HC).

C-NT1 and A-NT1 were recruited at the Centre for Narcolepsy of the Institute of Neurological Sciences of Bologna. Each patient enrolled in the present study received a diagnosis of NT1 according to the diagnostic criteria of the ICSD-3-TR [1], following a diagnostic protocol that included a clinical assessment by a neurologist, the Multiple Sleep Latency Test, hypocretin testing, and actigraphic assessment of the sleep/wake cycle. Each patient was drug-naïve at the time of actigraphic monitoring and received the first diagnosis of NT1 only at the end of the diagnostic protocol.

Both C-HC and A-HC were recruited at the Laboratory of Applied Chronopsychology of the University of Bologna. They underwent an anamnestic interview to exclude the use of psychopharmacological medication and drugs affecting sleep or activity. For the A-HC, shift work was used as an additional exclusion criterion.

Parents provided written informed consent for their children, while adults provided written informed consent before participating. Participants were originally enrolled in studies approved by the Ethics Committee of the Azienda Ospedaliero-Universitaria di Bologna (protocol no. 17009) and by the Ethics Committee of Rome (protocol no. 242/2024, approved on 18 November 2024).

### 2.2. Actigraphy

The actigraph model Micro Motiologger Watch (Ambulatory Monitoring, Inc., Ardsley, NY, USA) was used by each participant in the current study for an overall number of seven consecutive days and initialized through the Watchware software (version 1.99.34.1; Ambulatory Monitoring, Inc., Ardsley, NY, USA) to collect data of motor activity in epochs of 1 min. While the hardware consists of a piezoelectric accelerometer with a ≥0.01 g sensitivity, the sampling frequency is 10 Hz, and the filters are set to 2–3 Hz. Concurrently with actigraphic recording, participants were also asked to complete the sleep diary day by day, no later than 30 min after the last morning awakening.

The software Action W2 (version 2.7.3285; Ambulatory Monitoring, Inc., Ardsley, NY, USA) was used to score the actigraphic record using the algorithm by Cole and Kripke [13]. In more detail, an experienced scorer manually defined the start and end of each actigraphic night, taking into account the actigraph’s event-marker button, which participants pressed at lights-off and lights-on. If the participants forgot to press the event-marker button, the experienced scorer used the lights-off and lights-on times reported in the daily sleep diary to correctly set the time spent in bed.

The software Action 4 (Ambulatory Monitoring, Inc., Ardsley, NY, USA) was instead used to extract the raw motor activity counts, night-by-night and minute-by-minute, in the first 60 min after the get-up time, therefore describing the motor sleep inertia.

### 2.3. Activity-Based Prospective Memory Task

C-NT1, C-HC, A-NT1, and A-HC were requested to press the event-marker button on the actigraph at get-up time. In line with previous studies [14,15], this has been conceived as an ecological activity-based prospective memory task. We considered the task correctly performed only if the event-marker button was pushed no later than 60 min after get-up time, noting the time interval between these two moments (the response time) as a proxy for cognitive inertia. If the prospective memory task was not performed or was performed more than 60 min after get-up time, the corresponding awakening was discarded. The 60-min threshold was selected to be consistent with previous actigraphy-based ecological PM studies. Furthermore, we separated those who performed the PM task immediately at get-up time from those who performed it later using a 2-min threshold, based on the actigraph’s 1-min sampling frequency and the previous literature [11].

### 2.4. Statistical Analyses

First, an independent-samples Kruskal–Wallis test was performed with group (C-NT1, C-HC, A-NT1, A-HC) as the independent variable and response time as the dependent variable, with epsilon-squared (ε^2^) as the effect size measure. Pairwise comparisons were performed to explore the significant group effect.

Second, separately for children and adult samples, we carried out a chi-squared test to assess the distribution of NT1 and HC among the categories of compliant performers—those who remembered to push the event-marker button within 2 min from the get-up time—and late performers—those who pushed the event-marker button of the actigraph over 2 min from the get-up time. Cramér’s V as a measure of association strength was computed.

Third, separately for each of the four groups, we used the Functional Linear Modelling (FLM) [16] to examine in NT1 and HC the variation in the motor activity during the first 60 min after the get-up time according to the number of minutes between the get-up time and the moment participants remembered to push the event-marker button of the actigraph. Within the statistical framework of the FLM, two steps can be described. First, the raw motor activity pattern in the first 60 min (represented on the *x*-axis) after the get-up time was converted into a functional form by using the Fourier expansion model. Second, within that time interval, the non-parametric permutation F-test was adopted to verify whether and when the motor activity pattern differed significantly according to the time interval, computed in minutes, between the get-up time and the moment participants remembered to push the event-marker button of the actigraph—i.e., the response time—that was used as a continuous covariate. To perform the FLM, we used the “Actigraphy” package in R (R for Windows 3.5.3; RStudio 2022.07.1+554). In each FLM plot reported below (see the results Section 3.2), the upper panel shows the functional forms of the motor activity, with each line corresponding to a single participant, marked with a specific colour: the darker the colour, the shorter the response time, while the lighter the colour, the longer the response time. The lower panel shows the results of the non-parametric permutation F-test, with the red solid line pointing to the observed statistics. Meanwhile, the blue dashed and dotted lines indicate the global (most conservative) and point-wise (least conservative) tests of significance, respectively. Significant differences are observed when the red solid line is above the blue dashed line.

Each set of statistical analyses was carried out at the day level, i.e., considering each night of each participant.

## 3. Results

The C-NT1 group was composed of 21 participants (8 females and 13 males) with a mean age of 12.57 (SD = 3.53), while the C-HC group was composed of 20 participants (10 females and 10 males) with a mean age of 9.74 (SD = 0.93). As regards the adult samples, 31 A-NT1 patients (13 females and 18 males; mean age ± SD = 30.87 ± 15.08) and 51 A-HC (25 females and 26 males; mean age ± SD = 22.73 ± 3.12) were examined.

Since the number of valid awakenings ranged for each participant from 1 to 7, the analyses were conducted on a total number of 632 valid awakenings: C-NT1 = 168, C-HC = 90, A-NT1 = 205, A-HC = 169.

### 3.1. Activity-Based Prospective Memory Performance

**Figure 1:**
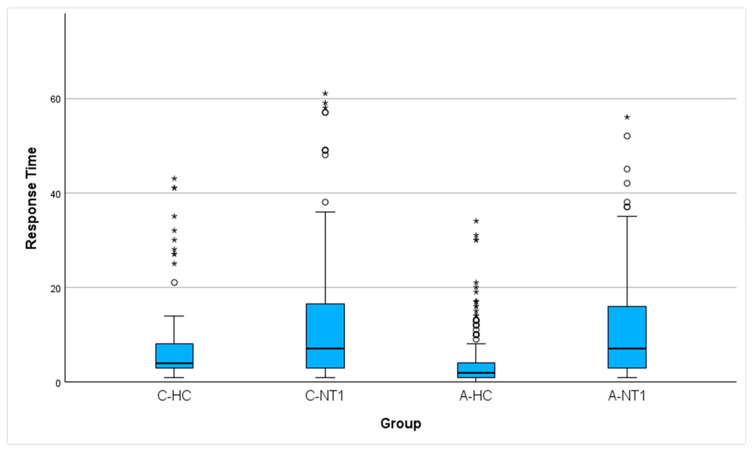
Boxplot of response time in C-HC (children healthy controls), C-NT1 (children patients with narcolepsy type 1), A-HC (adults healthy controls), and A-NT1 (adults patients with narcolepsy type 1). The box represents the interquartile range (IQR), which is the range between the first quartile (Q1) and the third quartile (Q3). The central line is the median (Q2). Whiskers (*) indicate the dispersion of values below the first quartile and above the third quartile, not classified as outliers (1.5 × IQR from the edge of the box). Circles (⚬) represent mild outliers (Q3 + 1.5 × IQR), and stars represent extreme outliers (Q3 + 3 × IQR).

With reference to the first statistical analysis, as reported in Figure 1, we observed a significant difference between groups in the response time (χ^2^_3_ = 138.09; *p* < 0.001; ε^2^ = 0.215).

The complete set of the results of the pairwise comparisons, performed to explore the significant group effect, is reported in Table 1. Notably, applying the Bonferroni correction for multiple tests, the time interval was similar between C-NT1 and C-HC, whereas it was significantly different between A-NT1 and A-HC.

**Figure 2:**
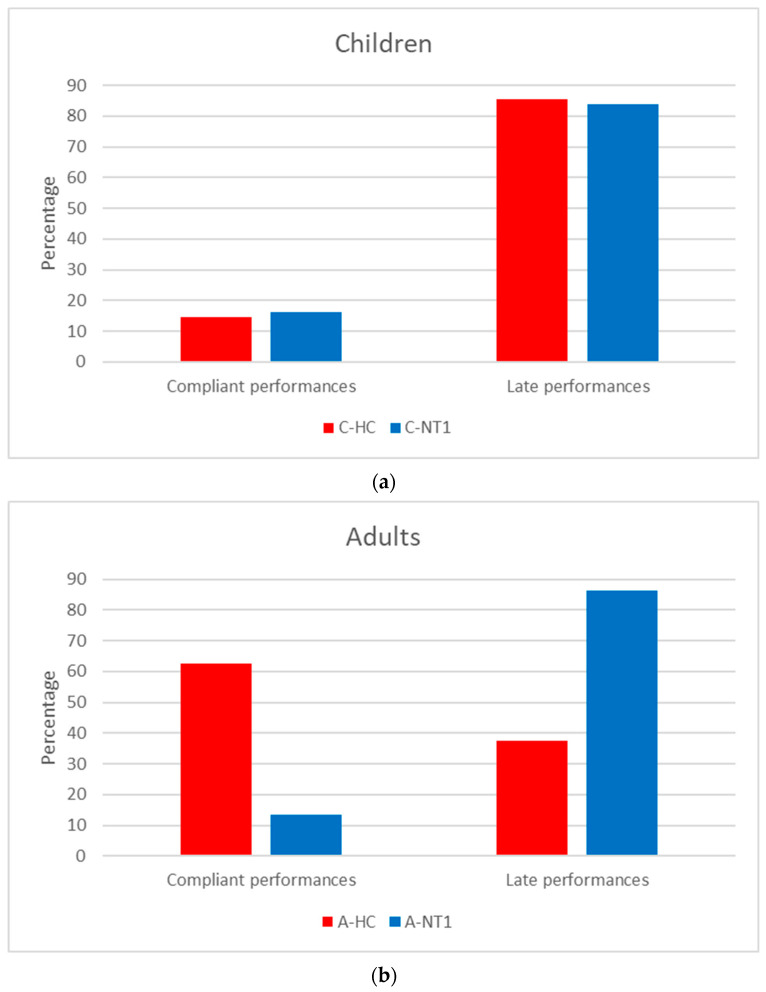
Distribution of NT1 and HC among the categories of late or compliant performances separately in children (**a**) and adults (**b**). Percentage values are shown.

As regards the second statistical analysis, the distribution of C-NT1 and C-HC among the categories of compliant and late performers was similar (χ^2^_1_ = 0.12; *p* = 0.73; V = 0.021) (Figure 2a) but significantly different in adults (χ^2^_1_ = 91.75; *p* < 0.001; V = 0.495) (Figure 2b), with a markedly low presence of A-NT1 among the compliant performers.

### 3.2. Motor Sleep Inertia and Activity-Based Prospective Memory Performance

#### 3.2.1. Children’s Populations

**Figure 3:**
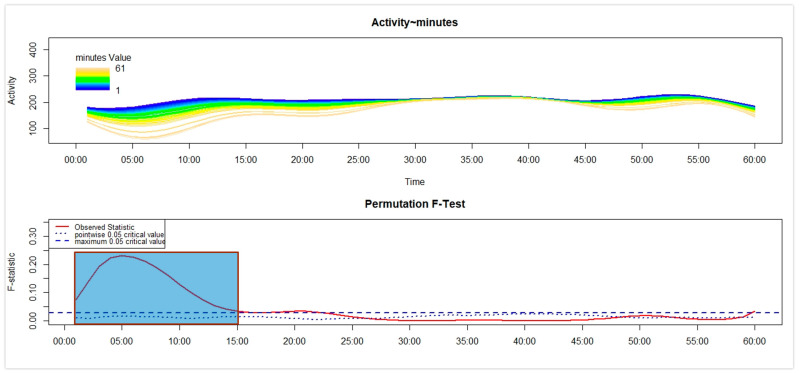
Functional linear modelling (FLM) applied to the analysis of the variation in the motor activity counts in C-NT1 in the first 60 min after the get-up time according to the response time—the time interval between the get-up time and the moment the person remembered to push the event-marker button of the actigraph. Significant differences are highlighted in the graph in blue.

In the sample of C-NT1 patients, as shown in Figure 3, higher motor activity in the first 15 min after the get-up time was significantly associated with a shorter response time.

**Figure 4:**
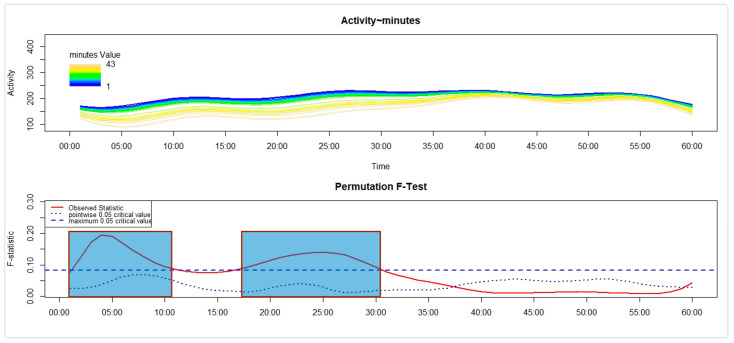
FLM applied to the analysis of the variation in the motor activity in the first 60 min after the get-up time in C-HC according to the duration of response time. Significant differences are highlighted in the graph in blue.

With reference to C-HC (Figure 4), we observed a significant association between higher motor activity and shorter latency in the execution of the activity-based prospective memory task across two time intervals: the first 11 min and the interval between 17 and 31 min after the get-up time. 

#### 3.2.2. Adult Populations

**Figure 5:**
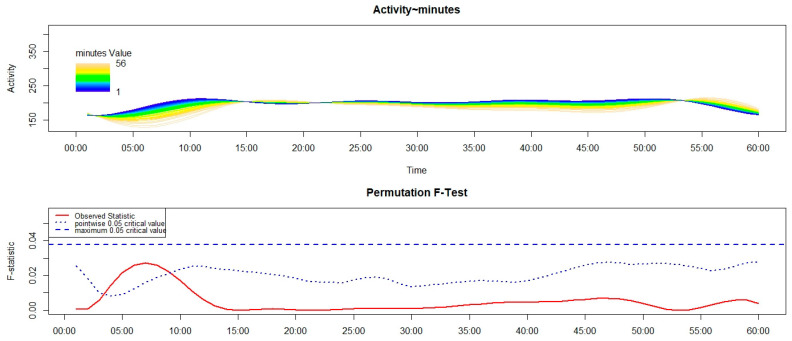
FLM carried out in A-NT1 to examine the variation in the motor activity in the first 60 min after awakening according to the duration of the response time.

With reference to A-NT1 (Figure 5), no significant associations between motor activity and the latency in the execution of the activity-based prospective memory task were observed.

**Figure 6:**
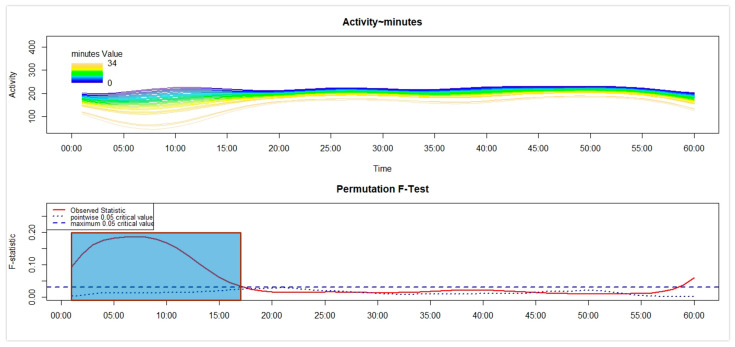
FLM performed in A-HC to explore the variation in the motor activity in the first 60 min after awakening as a function of the extent of the response time. Significant differences are highlighted in the graph in blue.

As regards A-HC (Figure 6), higher motor activity was significantly related to a shorter response time in the first 15 min after the get-up time.

## 4. Discussion

Regarding the results observed in the children’s samples, no significant difference was found between C-NT1 and C-HC in the time taken to perform the activity-based prospective memory task at awakening (Figure 1 and Table 1). Moreover, the distribution of C-NT1 and C-HC was not significantly different between compliant and late performers (Figure 2a). Lastly, a significant association was highlighted between higher motor activity at awakening and a shorter time taken to perform the memory task in both C-NT1 (Figure 3) and C-HC (Figure 4), although with a longer time interval in the latter group.

As regards the adults’ samples, A-NT1 took significantly longer to perform the memory task than A-HC (Figure 1 and Table 1). Furthermore, a significantly low number of A-NT1 was observed among compliant performers (Figure 2b). Finally, a significant association was observed between higher motor activity upon awakening and lower time to perform the memory task, but only in A-HC (Figure 6).

Overall, the impairment in the activity-based prospective memory performance (Figure 2) observed in A-NT1, a finding in line with previous studies that used a different actigraphy device [6,7], was not highlighted in C-NT1; thus, we would be able to propose a tentative answer to the question by Cano et al. [2] about the age-related changes in cognition in NT1, by underlying that the prospective memory impairments seem to be appreciated only in adult age. Moreover, while a significant relationship was detected between cognitive and motor inertia in C-NT1 (Figure 3), with minor cognitive inertia associated with lower motor sleep inertia, potentially interpreted as the by-product of a higher degree of synchrony between cognitive and motor systems, the same association was not observed in A-NT1 (Figure 5), underling once more as the C-NT1 population does not seem to present a prospective memory deficit.

Moving on to the interpretation of the results, a first working hypothesis can be proposed. More specifically, it may be suggested that C-NT1 can still employ some unaware and compensatory strategies that may prevent them from exhibiting the prospective memory failures observed in adult patients. A second working hypothesis is that higher neuronal plasticity in children may serve as a protective factor against impairment in prospective memory performance [17]. 

We also wish to add a further consideration on the lack of significant differences between C-HC and C-NT1. In both groups, the rate of compliant performances was significantly lower than that of late performances (Figure 2a). This finding can be understood in the context of the developmental trajectories of executive functions [18,19]. In fact, the development of prospective memory is significantly influenced by the growth of executive functions, which are linked to the development of the prefrontal cortex. Executive functions comprise three distinct but related cognitive processes: updating, which involves manipulating information stored in working memory; inhibition, which enables individuals to avoid automatic responses and resist distractions; and shifting, which facilitates transitions between tasks or mental sets [20]. Within a prospective memory task, these processes support the accurate retrieval of the intention and its execution at the appropriate time and in the appropriate manner. It can therefore be hypothesized that developmental age is particularly sensitive to the synchronization of these mechanisms, and that any misalignment among them may result in deficits in prospective performance [21].

To fully understand whether such explanations are sound, future longitudinal studies, ideally from childhood to adulthood, are highly recommended to track age-related changes in activity-based prospective memory performance.

With reference to a potential applied implication, by taking into account the specificity of the cognitive impairment in adult patients with narcolepsy and its potential effects on therapy adherence [5] and health-related outcomes, some strategies could be proposed at the time of beginning of pharmacological treatment as the encoding strategy, that was shown to be promising in healthy older adults and persons with very mild Alzheimer’s diseases to improve prospective memory performance [22].

Among the limitations of the current study, we may cite its exploratory nature, the cross-sectional design, and an unbalanced gender and age distribution between NT1 and HC in both children and adult samples. Moreover, we are unable to exclude the possibility that the ecological task may capture not only prospective memory per se, but also the structure of the morning routine, parental prompting in children, and motivational factors. This is not only not a limitation, but it actually reflects the very nature of prospective memory tasks, whose main characteristic is that they are embedded within an ongoing activity. For these reasons, PM is considered to be more than just memory [23]; it must also account for principles related to other cognitive processes, such as attention, time-related processes, and executive functioning.

## 5. Conclusions

The current study highlighted a poor activity-based prospective memory performance in A-NT1, while no significant impairment was observed in C-NT1. Moreover, contrary to C-NT1, the association between low cognitive inertia (i.e., short time interval between get-up time and the moment the activity-based prospective memory task was performed) and less motor sleep inertia (in other words, high motor activity after the awakening) was not observed in A-NT1 (i.e., a potential poor synchrony between cognitive and motor systems), therefore strengthening the proposal of strategies (e.g., encoding strategy) addressed to A-NT1 to improve prospective memory.

## Figures and Tables

**Table 1:** Statistics of the pairwise comparisons aimed at exploring the significant group effect on the time interval.

SAMPLE 1–SAMPLE 2	TEST STATISTICS	STD. ERROR	STANDARD TEST STATISTIC	SIG. ^A^
**A-HC–C-HC**	133.12	22.99	5.79	<0.001
**A-HC–C-NT1**	184.24	18.92	9.74	<0.001
**A-HC–A-NT1**	193.11	18.89	10.22	<0.001
**C-HC–C-NT1**	−51.12	23.75	−2.15	0.19
**C-HC–A-NT1**	−59.99	23.72	−2.53	0.07
**C-NT1–A-NT1**	8.87	19.81	0.45	1

^A^ Significant values have been adjusted by the Bonferroni correction for multiple tests. C-HC = children healthy controls; C-NT1 = children patients with narcolepsy type 1; A-HC = adults healthy controls; A-NT1 = adults patients with narcolepsy type 1.

## Data Availability

The data are unavailable due to privacy restrictions.

## Abbreviations

The following abbreviations are used in this manuscript:

ICSD-3-TR	The text revision of the third edition of the International Classification of Sleep Disorders
NT1	Narcolepsy type 1
HC	Healthy controls
SD	Standard deviation
FLM	Functional linear modelling
A-HC	Adult healthy controls
A-NT1	Adult with narcolepsy type 1
C-HC	Children healthy controls
C-NT1	Children with narcolepsy type 1
IQR	Interquartile ranges
Q1	First quartile
Q2	Second quartile
Q3	Third quartile
PM	Prospective memory
PMT	Prospective memory task
